# Ploidy influences cellular responses to gross chromosomal rearrangements in *saccharomyces cerevisiae*

**DOI:** 10.1186/1471-2164-12-331

**Published:** 2011-06-28

**Authors:** Paul P Jung, Emilie S Fritsch, Corinne Blugeon, Jean-Luc Souciet, Serge Potier, Sophie Lemoine, Joseph Schacherer, Jacky de Montigny

**Affiliations:** 1 Department of Genetics, Genomics and Microbiology, University of Strasbourg, CNRS, UMR7156, Strasbourg, France; 2 European Molecular Biology Laboratory, 69117 Heidelberg, Germany; 3 École normale supérieure, Institut de Biologie de l'ENS, IBENS, U1024 Inserm, UMR8197 CNRS, Paris, France

## Abstract

**Background:**

Gross chromosomal rearrangements (GCRs) such as aneuploidy are key factors in genome evolution as well as being common features of human cancer. Their role in tumour initiation and progression has not yet been completely elucidated and the effects of additional chromosomes in cancer cells are still unknown. Most previous studies in which *Saccharomyces cerevisiae *has been used as a model for cancer cells have been carried out in the haploid context. To obtain new insights on the role of ploidy, the cellular effects of GCRs were compared between the haploid and diploid contexts.

**Results:**

A total number of 21 haploid and diploid *S. cerevisiae *strains carrying various types of GCRs (aneuploidies, nonreciprocal translocations, segmental duplications and deletions) were studied with a view to determining the effects of ploidy on the cellular responses. Differences in colony and cell morphology as well as in the growth rates were observed between mutant and parental strains. These results suggest that cells are impaired physiologically in both contexts. We also investigated the variation in genomic expression in all the mutants. We observed that gene expression was significantly altered. The data obtained here clearly show that genes involved in energy metabolism, especially in the tricarboxylic acid cycle, are up-regulated in all these mutants. However, the genes involved in the composition of the ribosome or in RNA processing are down-regulated in diploids but up-regulated in haploids. Over-expression of genes involved in the regulation of the proteasome was found to occur only in haploid mutants.

**Conclusion:**

The present comparisons between the cellular responses of strains carrying GCRs in different ploidy contexts bring to light two main findings. First, GCRs induce a general stress response in all studied mutants, regardless of their ploidy. Secondly, the ploidy context plays a crucial role in maintaining the stoichiometric balance of the proteins: the translation rates decrease in diploid strains, whereas the excess protein synthesized is degraded in haploids by proteasome activity.

## Background

Gross chromosomal rearrangements (GCRs) lead to chromosomal instability and can enable organisms to adapt to new environments. However, this instability is also present in cancer cells: 90% of solid tumours have an abnormal number of chromosomes, a situation known as aneuploidy [1,2]. The presence of additional chromosomes is known to be characteristic of these cells but their role in cancerogenesis and tumour progression is poorly understood. The issue of their impact on cancer cells is still a matter of debate [3]. The implementation of aneuploidy can result from previous mutations in key genes involved in mitotic checkpoints such as *MAD2, BUB3 *or *BUBR1 *[4-7]. The baker's yeast *Saccharomyces cerevisiae *provides a suitable model for investigating the mechanisms responsible for GCRs and their effects on cellular physiology because it is easy to handle and shows a conveniently fast growth rate.

GCRs are infrequent spontaneous events and their selection in *S. cerevisiae *requires the use of genetic screening procedures, some of which are based on the responses of extracellular compounds such as drugs or on nutrient depletion [8,9]. In other cases, meiotic induction of strains with odd ploidy or diploid strains incapable of nuclear fusion has been performed to isolate haploid cells with one or more additional chromosomes [10,11]. Surveys on the cellular physiology of these strains showed the presence of growth rate defects linked to a cell cycle delay occurring during phases G1 and G2 [10,12]. The slowing of the cell cycle gives rise to abnormal cellular phenotypes showing an abnormally large intracellular volume or the formation of elongated buds [12]. Despite these negative effects on cellular fitness and morphology, GCRs can yield selective advantages under some environmental conditions (in the presence of rapamycin or bleomycin, for example) [10,11]. GCRs also have considerable effects at the molecular level, in terms of the correlation between copy number variations (CNV) and transcription [10,13]. The results recently obtained using proteomic approaches on aneuploid strains showed the existence of correlations between CNV, transcription and translation [11,14]. This conclusion is consistent with the hypothesis put forward by Torres and al [15]. that cellular responses may occur at translational rather than transcriptional level. Proteins involved in macromolecular complexes such as ribosomes and nucleosomes have to be stoichiometrically stable to prevent growth defects [16,17]. There are two hypotheses accounting for protein equilibrium in aneuploid cells: (i) the activity of the translational machinery may decrease or (ii) the excess proteins may be degraded shortly after being synthesized [15]. In disomic haploid strains of *S. cerevisiae*, mutation of the *UBP6 *gene, which enhances the proteasome activity, leads to a decrease in the doubling time [14]. This property suggests that the degradation of proteins is responsible for maintaining the protein balance in these cells. However, most previous studies have been performed on haploid strains, whereas little is known so far about the effects of GCRs on diploid cells.

To investigate the effects of the same GCRs in both haploid and diploid contexts, studies were performed on mutant strains of *S. cerevisiae *selected using a genetic screening method based on a mutated allele of the *URA2 *gene [18]. Several types of GCRs were selected depending on the ploidy context. Although aneuploidy and nonreciprocal translocation have been isolated in diploid strains, single gene duplication occurs only in haploids [19-21]. By contrast, segmental duplication and deletion have been described in both contexts [21-23]. To analyze the effects of ploidy on cellular responses, haploid and diploid revertants carrying the same GCRs were selected by inducing meiosis in diploid mutants or crossing haploid revertants with a strain bearing the *URA2 *mutated allele. Various approaches were used to study the effects of these GCRs on cells, such as changes in the colony and cell morphology as well as in the growth rates. These features showed that the fitness of mutants was impaired in comparison with the parental strains. In addition, transcriptomic analysis based on microarray hybridizations showed that many of the genes involved in ribosome biogenesis and translation mechanisms are down-regulated in all diploid revertants but up-regulated in haploids. In the haploid strains studied here, several genes involved in proteasome activity and its regulation are also up-regulated. These transcriptomic data suggest the existence of two distinct responses, depending on the ploidy context. In the presence of the same chromosomal rearrangement, diploid strains show a slower translation rate, whereas haploids help to conserve the cellular protein balance by degrading the excess protein synthesized.

## Results

### Collection of the studied strains

**Figure 1:**
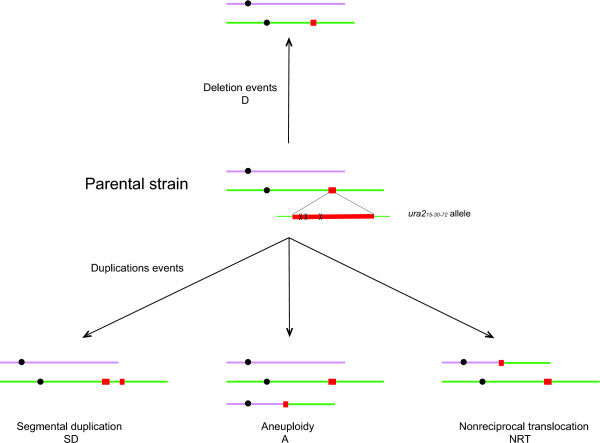
**The various types of GCRs selected using the positive selection screening method based on the *URA2 *gene**. Green rectangles stand for chromosome X, whereas pink rectangles indicate another chromosome. Black circles stand for centromeres and red squares for the *URA2 *gene. This gene contains 3 mutations (indicated by crosses) responsible for auxotrophy towards uracil.

The *URA2 *gene of *S. cerevisiae*, which is located on the chromosome X, encodes a multifunctional protein occurring in the pyrimidine biosynthesis pathway. This protein is composed of 3 active domains: glutamine amidotransferase (GATase), carbamoylphosphate synthetase (CPSase) and aspartate transcarbamoylase (ATCase). Three point mutations located in the proximal region of the *ura2_15-30-72 _*allele (Figure 1) lead to a loss of functions in the Ura2 protein and to auxotrophy towards uracil [18]. This auxotrophy is due only to the lack of ATCase activity, since the missing GATase and CPSase functions are compensated for by two isoenzymes present in the arginine biosynthesis pathway. Only ATCase reactivation is therefore required for prototrophic reversion to occur and it has been previously established that this reactivation is possible via complex chromosomal rearrangements [18-23].

In this study, we analyzed 10 diploid and 3 haploid revertants, which were previously isolated. These mutants carry deletions and duplication events of various kinds (Table 1). Duplications can be classified in 3 different groups: segmental duplications, nonreciprocal translocations and aneuploidies. In the case of segmental duplication (SD), the ATCase region and several adjacent genes are duplicated (Figure 1). Nonreciprocal translocation (NRT) events correspond to the duplication of the left part of the chromosome × (starting with the *URA2 *gene and extending up to the telomere) as well as the deletion of part of a chromosomal arm encompassing a telomere. This event leads to the formation of a chimeric chromosome, which sometimes forms an extra chromosome leading to aneuploidy (A) (Figure 1).

**Table 1:** Description of strains studied

Strain	Ploidy	GCRs	Duplicated region	Deleted region	Reference
A1-2n*	2n	Aneuploidy 2n+1	YJL130c-YJL225c YLR109w-YLR001c + YLL001w-YLL064c		[20]
A2-2n*	2n	Aneuploidy 2n+1	YJL130c-YJL225c YNL064c-YNL001w + YNR001c-YNR076w		[20]
A2-n*	n	Aneuploidy n+1	YJL130c-YJL225c YNL064c-YNL001w + YNR001c-YNR076w		In this study
A3-2n*	2n	Aneuploidy 2n+1	YJL130c-YJL225c YGL008c-YGL001c + YGR001c-YGR296w		[20]
A3-n*	n	Aneuploidy n+1	YJL130c-YJL225c YGL008c-YGL001c + YGR001c-YGR296w		In this study
A4-2n	2n	Aneuploidy 2n+1	YJL130c-YJL225c YLR249w-YLR001c + YLL001w-YLL064c		[20]
A4-n*	n	Aneuploidy n+1	YJL130c-YJL225c YLR249w-YLR001c + YLL001w-YLL064c		In this study
A5-2n*	2n	Aneuploidy 2n+1	YJL130c-YJL225c YMR186w-YMR001c + YML001w-YML132w		[20]
A5-n	n	Aneuploidy n+1	YJL130c-YJL225c YMR186w-YMR001c + YML001w-YML132w		In this study
NRT1-2n	2n	Non reciprocal translocation	YJL130c-YJL225c	YML186w-YMR326c	[20]
NRT2-2n	2n	Non reciprocal translocation	YJL130c-YJL225c	YHR174w-YHR219c-A	[20]
SD1-2n*	2n	Segmental duplication	YJL130c-YJL133c-A		[20]
SD1-n*	n	Segmental duplication	YJL130c-YJL133c-A		In this study
SD2-2n	2n	Segmental duplication	YJL130c-YJL151c		In this study
SD2-n*	n	Segmental duplication	YJL130c-YJL151c		[23]
SD3-2n*	2n	Segmental duplication	YJL130c-YJL190c		In this study
SD3-n	n	Segmental duplication	YJL130c-YJL190c		[23]
D1-2n*	2n	Deletion		YJL130c-YJL128c	In this study
D1-n*	n	Deletion		YJL130c-YJL128c	[22]
D2-2n	2n	Deletion		YJL130c-YJL076w	[21]
D3-2n*	2n	Deletion		YJL130c-YJL123c	[21]

Revertants used to transcriptomic analysis are indicated by an asterisk.

To investigate the effects of the same GCRs on cellular behaviour in haploid and diploid strains, revertants were constructed in both contexts. Unfortunately, haploid strains could not be generated from diploids carrying segmental deletions and nonreciprocal translocations. In fact, these rearrangements involve essential genes, which result in non-viable haploid strains [20,21]. Meiosis was therefore induced in the diploid mutant strains carrying an aneuploidy or a segmental duplication. After sporulation, segregants were grown in minimal medium and spores still carrying the GCR were selected. The ura^+ ^spores were conserved for further analysis. On the other hand, haploid revertants were crossed with a strain possessing the *ura2_15-30-72 _*allele in order to obtain diploid strains. This yielded a final set of 21 revertants, 16 of which carried the same GCRs in two different genetic backgrounds (Table 1).

### Ploidy content generates different colony morphologies

**Figure 2:**
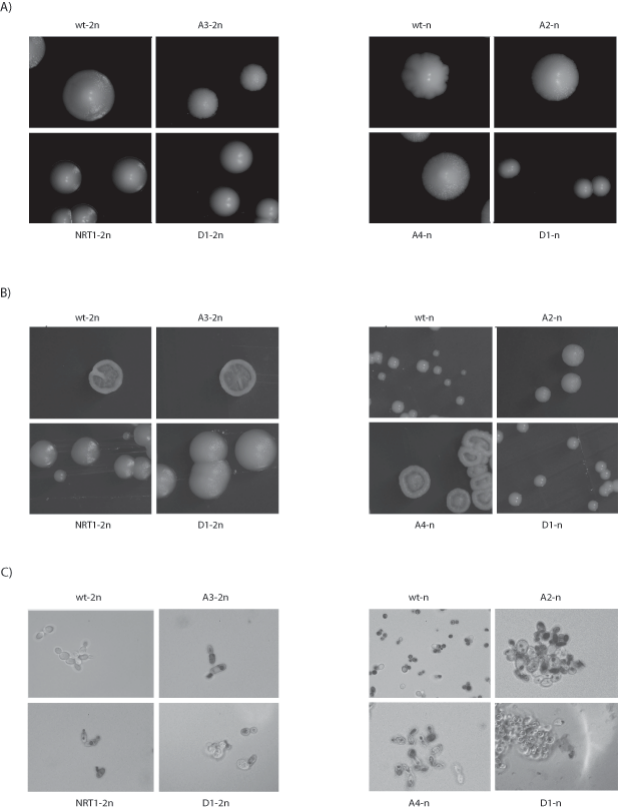
**Revertants showing different morphologies from the parental strains**. Diploid strains are on the left of the figure, whereas haploids are on the right. At macroscopic level, the variations were detected in the presence of glucose (A) were more striking with glycerol (B) (magnification × 10). At microscopic level (C), the neutral red staining observed shows that diploid and haploid parental cells (denoted wt-2n and wt-n, respectively) have classical ellipsoidal shapes, whereas revertants are more elongated and form aggregates (magnification × 1000).

The colony morphology of laboratory strains of *S. cerevisiae *is generally smooth whereas those isolated from nature and vineyards show a structured fluffy pattern [24]. However, the same strain can also show various morphological features, depending on the carbon source used [25]. Mutant and parental strains were plated onto a solid YPD medium containing 2% glucose. In the haploid context, the colony morphology was found to be of two kinds in the revertant A5-n strain: one with a smooth appearance and one with a granular appearance (see Additional file 1, Figure S1). When these two colonies were spread on minimum medium containing no uracil in order to maintain the selection pressure, only granular colonies developed. This suggests that the additional chromosome essential to uracil prototrophy is unstable in this mutant. This chromosome is therefore conserved only in cells generating granular colonies. By contrast, colony morphology of other revertants showed only one homogeneous type of phenotype differing from the parental strains. For example, the haploid parental strain has an irregular shape whereas the mutants can be either circular and smooth (D1-n, SD1-n) or circular and rough (A2-n, A3-n, A4-n) (Figure 2 and Figure S 2).

The use of other carbon sources such as glycerol (3%), galactose (2%), sorbitol (2%), ethanol (2%) and acetic acid (2%) brought to light the existence of some more striking discrepancies between the mutant and parental strains. Differences were observed, for example, between the NRT1-2n revertant and the parental diploid strain in the presence of glycerol. The parental strain has peripheral swellings with a central invagination, whereas NRT1-2n strain is circular with a fluffy appearance (Figure 2 and see Additional file 2, Figure S2).

These comparisons also showed the existence of morphological differences between revertants carrying the same GCRs, depending on the ploidy context. As shown in Figure 2B, colonies of the revertant D1-n were found to be smaller than the D1-2n colonies, which suggests that the ploidy has decisive effects on the cellular responses. In addition to these morphological findings, the patterns of growth on non-fermentable substrates such as glycerol and ethanol showed that the revertants' respiration capacity was preserved (see Additional file 2, Figure S2).

### GCRs generate abnormal cellular morphologies

It was established a decade ago that the increase in ploidy level from haploidy to tetraploidy results in an increase in the intracellular volume, which induces cell shape modifications [26]. However, the presence of GCRs can also give rise to abnormal cellular morphological features such as pseudohyphal growth, multibudded cells and cells with elongated buds [12,27]. With a view to comparing the cellular morphology of revertants and parental strains, cells were grown in minimum liquid medium with 2% glucose up to mid-log phase and examined under light microscopy at a magnification of × 1000. Haploid and diploid parental strains both showed a classical ellipsoidal shape, but not the mutants (Figure 2). As shown in Figure 2C, revertants A2-n and D1-n formed aggregates, whereas A3-2n and A4-n were more elongated than the parental strains, which suggests that an increase in the cellular volume had occurred. This increase is consistent with the delayed G1 phase observed in haploid disomic strains [10]. This time lag may be due to the regulation of the 17 genes thought to be involved in the cellular volume increase in *S. cerevisiae *such as the G1 cyclins encoded by the *CLN1 *and *PCL1 *genes or *FLO11 *[26,28]. The presence of an additional chromosome or GCRs may impair the molecular pathway, leading to the deregulation of several genes including cyclins and *FLO11*.

### Growth defects are more obvious in diploid revertants

**Figure 3:**
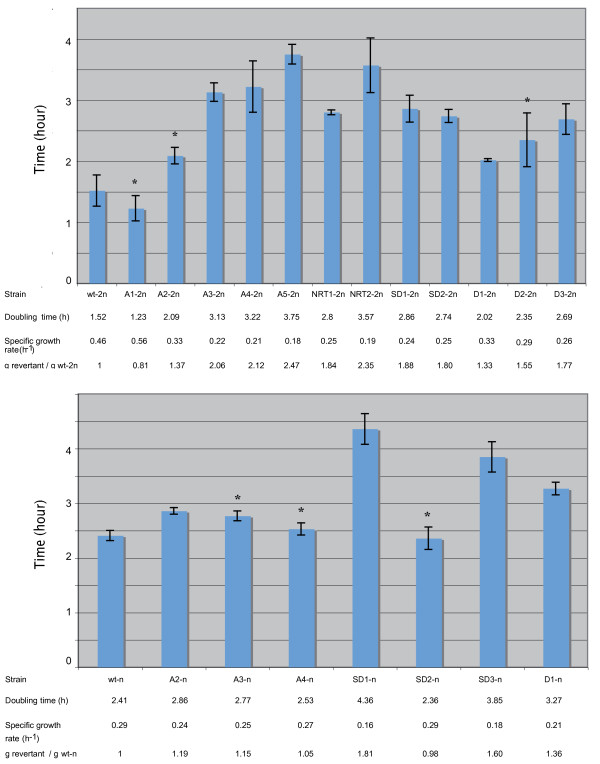
**Doubling time and specific growth rates**. Diploid strains are shown at the top of the figure and haploid strains at the bottom. Asterisks indicate values that are not significantly different from those obtained on parental strains. In each case, the parental strain is presented on the left of the graph and denoted wt-2n and wt-n.

To analyze the effects of GCRs on the doubling time, the specific growth rates of cells grown in minimum medium were determined during the exponential growth phase. All experiments were performed in triplicate. Student's T-tests were performed on the results obtained. Revertants giving a p-value < 0.05 were taken to have a different doubling time from that of the parental strains. The diploid parental strain has a doubling time equal to one division every 1.5 hour, whereas all the revertants except A1-2n, A2-2n and D2-2n have longer doubling times. In the main diploid mutant strains studied, the specific growth rate was even found to be twice as fast as that of euploid strains (Figure 3). In the haploid context, parental strain buds every almost 2.5 hours, and the doubling time increased by a factor of less than two in the case of only three revertants (SD1-n, SD3-n and D1-n) (Figure 3). To compare the effects of GCRs depending on the ploidy, the ratios between the doubling times of revertant and parental strains (g_revertant_/g_wt_) were determined. Comparisons between these ratios showed that with the same GCRs, this ratio was almost equal to 2 in case of diploid revertants but only about 1 in that of haploids, which suggests that GCRs generate two distinct responses, depending on the ploidy.

### Metabolic properties and ATP production rates are impaired

**Figure 4:**
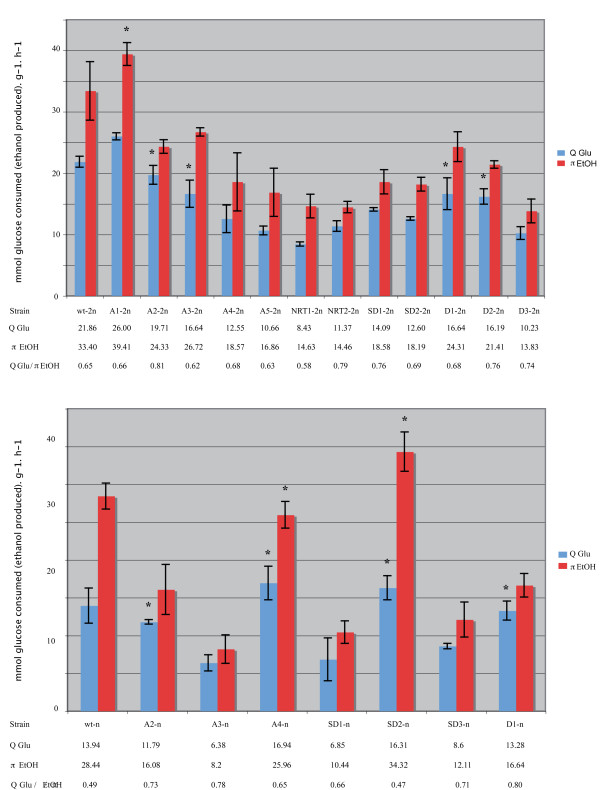
**Specific glucose consumption rate (Q**_**Glu**_**) and specific ethanol production rate (π**_**EtOH**_**)**. Diploid strains are presented at the top of the figure and haploid strains at the bottom. Asterisks indicate values that are not significantly different from those obtained on parental strains.

To determine the metabolic impairments involved, various properties, including the specific glucose consumption rate (Q_Glu_) and the specific ethanol production rate (π_EtOH_), were studied. These parameters were defined as the amount of glucose consumed and the amount of ethanol produced per hour and per gram of biomass generated. As previously, all theses experiments were again performed in triplicate. Student's T-tests were performed on the data obtained. The values of these parameters were found to differ between the parental and mutant strains at both ploidy levels. In diploid revertants, only A1-2n consumed glucose faster than the diploid parental strain, and the differences did not differ significantly in the case of A2-2n, A3-2n, D1-2n and D2-2n (Figure 4). Lower glucose consumption rates were observed in the last seven diploid revertants studied (Figure 4). In haploid strains, three revertants showed a decrease in Q_Glu_, whereas this rate did not differ significantly in the other haploid mutants in comparison with the parental strain (Figure 4). Likewise, π_EtOH _was found to decrease in all the mutants except for the diploid A1-2n and the haploids A2-n, SD1-n and SD3-n (Figure 4).

The Q_Glu_/π_EtOH _ratio can be used to assess the amount of ethanol produced per mmole of glucose consumed. A high Q_Glu_/π_EtOH _ratio means that a low amount of ethanol is produced. Classically, the alcoholic fermentation of 1 molecule of glucose results in the formation of 2 molecules of ethanol, as in the case of the haploid parental strain showing a ratio of 0.49. Higher ratios (of up to 0.80 in the case of D1-n), suggesting the occurrence of a decrease in the ethanol production rates, were obtained in most of the haploid revertants (Figure 4). In the diploid strains, Q_Glu_/π_EtOH _also increased in the five mutant strains A2-2n, NRT2-2n, SD1-2n, D2-2n and D3-2n, ranging from 0.74 to 0.81 (Figure 4). This decrease in the ethanol production rate per molecule of glucose uptake suggests that this sugar may be used differently in the mutants to achieve the ATP production rates required for cell survival and proliferation.

### Cellular response depends on the ploidy context

To determine the effects of GCRs on the patterns of gene expression, each pair of mutants and parental strains were grown in minimum liquid medium up to mid-log phase and a comparative transcriptomic analysis based on microarray hybridizations was conducted. Each experiment was performed in duplicate. Results obtained on 14 revertants were used for further analyses depending on the reproducibility of the replicates (Table 1). A global analysis of the transcriptomic data was performed on all the genes to define the proportion of genes that are differently regulated in mutants in comparison with the parental strains. Results showed that up to 2/3 of all the genes are misregulated in haploid mutants in comparison with the parental strain, whereas this proportion is less than 1/3 in diploids.

**Figure 5:**
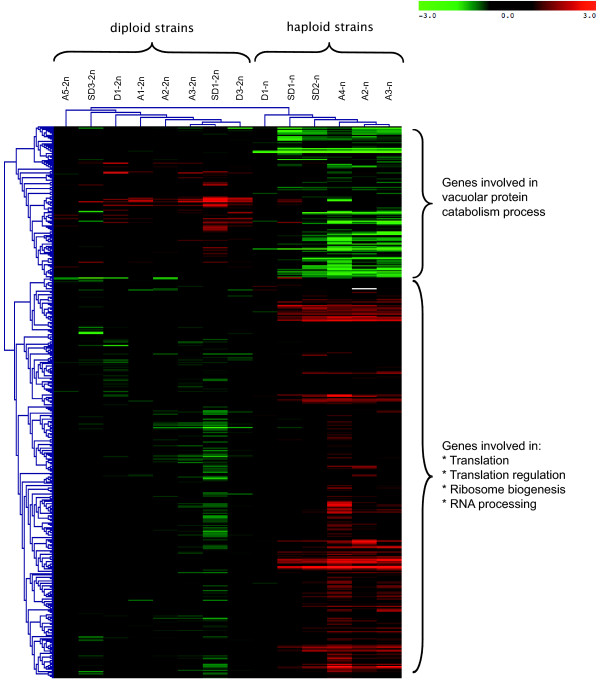
**Expression profile of regulated ESR genes**. This figure shows the hierarchical clustering of the 457 statistically regulated genes. Up-regulated genes are depicted in red and down-regulated genes in green.

We clustered misregulated genes in different groups depending on the ploidy and the type of GCRs. Each group was then divided into 2 subgroups depending on whether these genes were up- or down-regulated. To identify the functional categories that were enriched in the various subgroups, the GO Term Finder program was used [29]. In all the diploid and haploid revertants studied, a similar pattern of up-regulation was observed among the genes involved in energy metabolism, such as those encoding subunits of the succinate dehydrogenase complex and those encoding proteins involved in the tricarboxylic acid cycle. This finding is consistent with the fact that higher ATP production rates were observed in revertants than in the parental strains. However, some differences were also found to exist between haploid and diploid revertants showing exactly the same GCRs. Genes involved in the composition of the ribosome and in RNA processing were found to be down-regulated in diploids but up-regulated in haploids (Figure 5). In addition, only haploids showed over-expression of genes involved in the regulation of the proteasome, which suggests the occurrence of hyperactivity in this complex. All in all, these data suggest not only that the cellular responses depend on the ploidy level, but also that both haploid and diploid strains require to be provided with additional energy supplies to be able to proliferate.

### GCRs generate a stress response in both ploidy contexts

Almost 850 genes in *S. cerevisiae *have been described as environmental stress response elements (ESR) and are known to be involved in carbohydrate metabolism or protein metabolism [30]. A t-test of these stress response genes in all the revertants studied here showed that 457 of these genes are misregulated. The most noteworthy result obtained was that ESR genes are regulated differentially, depending on their ploidy context (Figure 5). As mentioned above, many genes involved in translation and ribosome biogenesis are differentially expressed in revertants. These genes are down-regulated in diploid strains and up-regulated in haploids (Figure 5). These results suggest that all GCRs induce a general stress response in these mutants, regardless of their ploidy.

One haploid revertant (D1-n) is especially interesting because only 223 of its genes are differentially expressed, whereas the average number of regulated ESR genes in haploid mutants is 408. This strain contains a deletion event at the locus where the ATCase region of *URA2 *is fused to the *PBS2 *gene. The protein encoded by this gene is a MAP kinase kinase involved in the HOG signalling pathway [31]. The phosphorylation of the Hog protein by Pbs2p results in the activation of transcription factors Msn2p and Msn4p, which regulate many ESR genes [32,33]. When the *PBS2 *gene is mutated, cells are more sensitive to the polymyxin B antibiotic [34]. The fact that D1-n is more sensitive to this antibiotic than the haploid parental strain shows that the kinase is not functional in this revertant. This finding helps to explain the existence of this small set of misregulated ESR genes. It also suggests that all the revertants respond to GCRs acting as environmental stresses. This response is partly independent of the HOG signalling pathway.

## Discussion

The DNA content of laboratory strains of *S. cerevisiae *is variable, and the nucleotide divergence ranges from 0.05% between A364A and S288c to 0.36% between SK1 and S288c [35]. The genetic background may have an impact on several cellular features such as the sporulation efficiency, the occurrence of GCRs and the colony morphology [25,36,37]. For example, genetic variations between SK1 and S288c strains result in different colony morphologies, ranging from a simple smooth colony in the case of S288c to a complex colony with a coralline aspect in that of SK1 [25]. The presence of GCRs changes the DNA content of a cell and hence its genetic background, but little is known so far about the effects of these rearrangements. The colony morphology of revertants observed on different substrates (glucose, galactose, sorbitol, ethanol, acetic acid and glycerol) varies considerably (Figure 2 and see Additional file 2, Figure S2). In addition, the fact that the colony phenotypes of revertants carrying the same GCRs in the haploid and diploid contexts also show considerable differences suggests that ploidy plays a crucial role in the cellular responses. Microscopic studies on cell shapes have also shown the existence of differences between parental and mutant strains. The parental cells have a classical ellipsoidal form, whereas the revertant cells are more elongated and can form aggregates. These changes may have resulted from an increase in intracellular volume that is proportional to the ploidy in *S. cerevisiae *[26]. One possible explanation for this increased cellular volume is that a cell cycle delay may have occurred, as in disomic strains of *S. cerevisiae *[10].

The doubling times of most of the diploid revertants were much greater than those observed in the parental strain. In the haploid context, however, the growth rates seem to be less affected than in diploids because only half of the revertants showed longer doubling times. The variability of these doubling times may be due to cell cycle delays resulting from the mutation of key proteins such as the G1 cyclins encoded by *CLN1 *and *PCL1 *genes or the subunit of the conserved heterohexameric MCM2-7 helicase encoded by *MCM4 *gene [26,38]. However, these cell cycle delays may also result from various global responses depending on the ploidy context.

To obtain further insights into the effects of ploidy on cellular responses, a comparative gene expression analysis was performed between mutant and parental strains. The results show that the number of misregulated genes range from 1/3 in diploid revertants to 2/3 in haploid mutant strains. GO Term analysis of these differently regulated genes indicated that genes involved in energy metabolism (in the tricarboxylic acid cycle and the oxidation phosphorylation pathway, for instance) are up-regulated in both contexts. This finding is consistent with the increase in the Q_Glu_/π_EtOH _ratio observed in most of the revertants studied here. All in all, these data suggest that less ethanol is produced per molecule of glucose consumed, probably in order to ensure that the ATP required for cell proliferation is available.

A higher ATP production rate may be required for the replication or the transcription of additional genes as well as for their translation and the possible degradation of unwanted proteins. However, this excess ATP may also constitute a cellular stress, as described in the case of disomic strains of *S. cerevisiae *and defined as an aneuploidy stress response [39]. Among the 850 or so ESR genes defined under various stress conditions [30], 457 are significantly misregulated in the haploid and diploid revertants, which suggests that all GCRs exert stresses on the cells. It has clearly emerged, however, that cellular responses depend on the ploidy context because of the symmetrical patterns of the misregulated genes between haploid and diploid strains. Indeed, when some genes are up-regulated in diploid revertants, they are down-regulated in haploids and *vice versa*. Analysis of these misregulated ESR genes showed that almost 330 of them, which are involved in ribosome biogenesis and RNA processing are down-regulated in diploid mutant strains but up-regulated in haploids. This finding suggests that the translation rate decreases only in diploids. The fact that the genes involved in proteasome regulation and activity are up-regulated only in haploid revertants suggests that the protein equilibrium may be preserved in two different ways depending on the ploidy context. Diploid revertants seem to decrease the translation rate, whereas haploids increase the protein degradation rate.

## Conclusion

The present comparisons on cell and colony morphology and fitness show that GCRs induced physiological impairments in all the revertants studied. It was also observed that in order to proliferate, all mutant strains require additional energy, the lack of which probably generates a stress response. The transcriptomic data obtained here suggest that the cellular responses depend on the ploidy level. To balance the protein stoichiometry, diploid revertants decrease the expression level of the genes involved in the translational mechanisms, whereas haploids increase the expression level of the genes involved in proteasome activity. These results also raise the question of how to prevent the proliferation of cells with GCRs, such as cancer cells. It would be interesting to obtained further insights into the direct contribution of the translational machinery as well as that of the proteasome activity to the fitness of both haploid and diploid mutants.

## Methods

### Strains and growth media

All the strains used in this study are isogenic to the laboratory strain FL100 and were obtained using the positive selection screening method described previously [19]. Yeast cells were grown at 30°C in liquid or solid (2% agar) yeast peptone (YP) with various sources of carbon and supplemented yeast nitrogen base (YNB).

### Strain construction

Diploid revertants were sporulated to isolate the ura^+ ^haploid strains. Haploid revertants were crossed with a strain isogenic to FL100 that possesses the *ura2_15-30-72 _*allele in order to obtain diploid cells with the chromosomal rearrangements required for prototrophy towards uracil.

### Characterization of colony morphology

Cell cultures were grown in YNB medium in mid-log phase (0.35 < OD_600 _< 0.45), placed on slides, stained with neutral red and examined by light microscopy (magnification × 1000). The colony morphology was determined after incubating the cells for 6 days on solid YP media with glucose (2%), galactose (2%), ethanol (2%), sorbitol (2%), glycerol (3%) or acetic acid (2%). Pictures were obtained using a Leica Z16 APO A Macroscope (magnification × 10).

### Fitness, glucose uptake and ethanol production

Cell cultures were grown in 50 ml of YNB medium, starting with a low OD_600 _= 0.002. After 12 hours of growth, samples were collected every 2 hours and the OD_600 _was measured to determine the growth rates. All samples were also used to determine the glucose uptake and ethanol production rates. Residual glucose rates and ethanol concentrations were determined using the D-Glucose kit and the Ethanol kit from R-BIOPHARM, respectively. Batch cultures were performed concomitantly in 200 ml of YNB medium to determine the dry weight of each strain. Cell cultures were collected in the mid-log phase and dried at 65°C for 2 days.

Specific glucose consumption rates (Q_Glu_) were determined during the exponential growth phase and expressed as the amount (in millimoles) of glucose consumed divided by the amount of biomass produced (in grams of dry weight) and multiplied by the corresponding specific growth rates (in terms of the increase in the biomass per hour). The specific ethanol production rates (π_EtOH_) were also determined and expressed as the amount of alcohol produced per hour per gram of biomass.

### RNA extraction and gene expression arrays

10^8 ^cells in the mid-log phase (0.33 < OD_600 _< 0.36) grown in YNB medium were harvested on filters, frozen in liquid nitrogen and conserved at -80°C until RNA preparation was performed. Filters were incubated at 65°C for 1 hour in lysis buffer (10 mM EDTA, 0.5% SDS and 10 mM Tris, pH 7.5) and acid phenol and then placed on ice for 10 min. The aqueous phase was further extracted with an equal volume of chloroform. Total RNAs were then precipitated overnight in the presence absolute ethanol and sodium acetate 4M. The RNA was then purified using RNeasy columns (Qiagen).

Quantity and integrity of the RNA were measured with the RNA 6000 nano assay kit using an Agilent 2100 bioanalyser (Agilent Technologies, Germany). 1.5 μg of total RNA was used for each reverse transcription reaction and indirectly labeled with Cy3 and Cy5, and subsequently hybridized on microarrays. Agilent DNA microarray slides (GE 8 × 15K n AMADID 015761) containing probes for most of the yeast open reading frames were used. Two hybridizations were performed for each comparison using the dye-switch procedure. The arrays were scanned using a Genepix 4000 scanner.

The full dataset can be downloaded from the Gene Expression Omnibus (GEO) database (http://www.ncbi.nlm.nih.gov/geo/) [40] under the accession series number GSE28285.

### Data analysis

The microarray data were normalized without performing background substraction, using the global Lowess method performed with the Goulphar software program [41]. Gene profile analyses were performed using the Java Treeview software program [42]. Hierchical clustering and Student's t-tests were performed using Pearson correlation with the MeV software program [43]. Gene ontology was analyzed using GO Term Finder http://go.princeton.edu/cgi-bin/GOTermFinder[29].

## Authors' contributions

Conceived and designed the experiments: JPP, JS, JdM. Performed the experiments: JPP, ESF, CB. Analyzed the data: JPP, SL, JS. Contributed reagents/materials: JLS, SP. Wrote the paper: JPP, JS. All authors read and approved the final manuscript.

## Supplementary Material

Additional file 1 **Figure S1 - Colony morphology of the revertant A5-n**. In the presence of glucose, two distinct colony morphologies were observed (as shown by the arrows). One type of colony is small and circular, whereas the other one is larger and has a granular appearance. Click here for file

Additional file 2 **Figure S2 - Colony morphologies**. Each revertant (diploid (A) and haploid (B)) was spread on medium containing various carbon sources: glucose, glycerol, galactose, sorbitol, ethanol and acetic acid. Conspicuous morphological differences were visible in these media, especially in the presence of the non-fermentable compounds such as glycerol and ethanol. For example, in the presence of glycerol, the diploid parental strain has peripheral swellings with a central invagination whereas NRT1-2n has a fluffy appearance. The shapes of the colonies are also variable: some of them are circular, whereas others are irregular, such as revertants A3-2n and A2-2n in the presence of glycerol. Click here for file
